# An Improved Compressive Sensing and Received Signal Strength-Based Target Localization Algorithm with Unknown Target Population for Wireless Local Area Networks

**DOI:** 10.3390/s17061246

**Published:** 2017-05-30

**Authors:** Jun Yan, Kegen Yu, Ruizhi Chen, Liang Chen

**Affiliations:** 1 College of Telecommunications and Information Engineering, Nanjing University of Posts and Telecommunications, Nanjing 210003, China; yanj@njupt.edu.cn; 2 School of Geodesy and Geomatics and the Collaborative Innovation Center for Geospatial Technology, Wuhan University, Wuhan 430079, China; kgyu@sgg.whu.edu.cn; 3 State Key Laboratory of Information Engineering in Surveying, Mapping and Remote Sensing, Wuhan University, Wuhan 430079, China; ruizhi.chen@whu.edu.cn; 4 Collaborative Innovation Center of Geospatial Technology (INNOGST), Wuhan 430079, China

**Keywords:** compressive sensing, positioning, received signal strength, target population, wireless local area network

## Abstract

In this paper a two-phase compressive sensing (CS) and received signal strength (RSS)-based target localization approach is proposed to improve position accuracy by dealing with the unknown target population and the effect of grid dimensions on position error. In the coarse localization phase, by formulating target localization as a sparse signal recovery problem, grids with recovery vector components greater than a threshold are chosen as the candidate target grids. In the fine localization phase, by partitioning each candidate grid, the target position in a grid is iteratively refined by using the minimum residual error rule and the least-squares technique. When all the candidate target grids are iteratively partitioned and the measurement matrix is updated, the recovery vector is re-estimated. Threshold-based detection is employed again to determine the target grids and hence the target population. As a consequence, both the target population and the position estimation accuracy can be significantly improved. Simulation results demonstrate that the proposed approach achieves the best accuracy among all the algorithms compared.

## 1. Introduction

With the wide deployment of the mobile wireless systems and networks, wireless positioning has recently drawn considerable attention [1,2,3]. Due to government regulations and commercial applications, the location based services (LBS) are made possible on laptops, smart phones and personal digital assistants (PDAs) [4,5]. However, typical Global Navigation Satellite System (GNSS) cannot perform well in urban canyons and indoor scenarios when GNSS signals are blocked. Thus, non-GNSS positioning systems are required for these scenarios and indoor positioning has become a hot research topic in recent years [6,7].

Currently available indoor positioning systems include cellular network-based systems [8,9,10,11], single frequency network-based systems [12,13,14,15], ultra-wide band (UWB)-based systems [16], wireless local area network (WLAN)-based systems [17,18], radio frequency identification (RFID)-based systems [19,20], dead-reckoning-based systems [21], Bluetooth indoor positioning systems [22] and pseudolite-based systems [23]. Among them, the received signal strength (RSS)-based WLAN indoor positioning system is the most popular technique employed [24,25,26,27]. In this paper, we will also focus on RSS-based indoor positioning.

In general, the RSS-based methods can be classified into two broad categories, namely the distance prediction-based and fingerprinting-based methods. Due to the complicated indoor environments, large errors occur when using a theoretical propagation model to characterize the distance between the emitter and the receiver [1]. Comparatively, the RSS fingerprinting method is able to achieve acceptable indoor positioning accuracy, however, to construct and maintain the signature map is a time consuming task [28]. Therefore, to develop alternative solutions is thus of great interest to overcome the drawbacks of the current RSS-based WLAN indoor positioning approaches.

Recently, compressive sensing (CS) theory has been used to provide a novel framework for sparse signal processing [29]. From the viewpoint of WLAN-based indoor positioning, the RSS vector measured from access points (AP) changes as a mobile device moves from one position to another. Also, the target population is usually much smaller than the number of discrete grids defined over the location area covered by the WLAN. Thus, the problem of WLAN-based positioning can be formulated as a sparse signal recovery problem. The target positions can be estimated in the discrete spatial domain by solving an under-determined linear system with a limited number of RSS measurements. CS technique positioning algorithms are typically able to achieve good accuracy and robustness when only sparse information is available [30].

In this paper, we focus on dealing with CS-based localization in the absence of prior knowledge of the target population. In particular, an effective two-phase localization algorithm is proposed. The major contributions of the proposed approach are two-fold.

The first contribution is the improved accuracy in target population estimation. In the coarse localization phase, by determining the signal recovery vector and employing the threshold- based detection, the initial candidate target grids are determined. In the fine localization phase, based on the updated measurement matrix, the signal recovery vector is re-estimated and the identification of the target grids is performed again to reject the outliers or false target grids produced in the coarse localization phase. As a result, the target population estimation is more reliable.

The second main contribution is the solution to the problem of a large target position error caused by large grid size. In the coarse localization phase, the initial candidate target grids are identified. In the fine localization phase, each of the candidate grids is divided into four equal grids in turn. Through iterative partition of a grid, the position of each target is constrained to a sufficiently small grid. As a consequence, the position accuracy can be improved greatly.

The remainder of this paper is organized as follows: Section 2 provides an overview of the related work. The CS-based positioning model when using RSS measurements is described in Section 3. The proposed two-phase positioning approach is presented with a detailed description in Section 4. Simulation results are reported in Section 5. Finally, Section 6 draws our conclusions.

## 2. Related Work

In the literature, a variety of methods and techniques have been proposed for RSS-based WLAN indoor positioning. In [31], a novel localization protocol was proposed to use the CS theory to reformulate the localization problem in wireless networks and the theoretical CS-based localization framework was described in detail. The authors of [32] provided a rigorous proof for the necessity of Restricted Isometry Property (RIP) and conducted a comprehensive analysis about how to choose the grid size. In [33,34], a two-step CS-based indoor positioning algorithm was proposed, which consists of a coarse localization by cluster matching and a fine CS-based localization. In the coarse localization step, the orthogonalization preprocessing procedure was used to induce incoherence needed in the CS theory. In fine localization step, the AP selection techniques were utilized to decrease computational complexity and increase accuracy. The authors of [35] extended the application scenario from a static target to a mobile one. Specifically, the location problem was solved by first applying a proximity constraint to limit the distance between a coarse estimate of the current position and a previous estimate. Then, a CS-based scheme was applied to obtain a refined position estimate by a map-adaptive Kalman filter. In [36], issues of signal-to-noise ratio (SNR), target position refinement, number of measurements, linear programming scheme, sensor deployment and grid topology were investigated for CS-based positioning; it was observed that noise power had the main effect on RSS based localization performance. In [37], the authors applied the CS technique to perform sparsity-based indoor localization. It was an energy-constrained algorithm which can reduce the amount of information transmitted from a wireless device with limited power, storage and processing capabilities to a central server. In [38], a data processing technique was proposed for CS-based and fingerprinting based indoor positioning by using the signal strength differential (SSD) measurements. To mitigate the influence of large measurement noise, a sparse transformation model based on Gaussian kernel function was proposed to transform the location vector into a strictly sparse one. Besides, in order to lower the high computational complexity, several fingerprinting space filtering algorithms were also exploited to remove some useless fingerprints in the radio map according to the real-time RSS observations.

From the above discussions, it can be seen that most of existing work on CS-based positioning focused on theoretical modeling and analysis [31,32,36], satisfied conditions for CS theory [33,34,35], computational complexity [33,34,38] and positioning performance [33,34,35,38]. One of the significant advantages of CS-based positioning is that positions of multiple targets can be simultaneously estimated, but most existing algorithms are based on the assumption of a single target [33,34,35]. Although there are a few multi-target positioning methods in the literature, the number of targets is always assumed known in advance [39]. In addition, some common CS recovery algorithms, such as BP algorithm [40] and OMP [41], also require a known degree of sparsity. In a real network-based-positioning system, the target population is usually an unknown variable. In [42], an unchanged residual error rule based method was proposed to determine the unknown target population. However, there is a significant issue when implementing such an approach, because it is difficult to choose an appropriate criterion to terminate the search procedure. In order to apply CS theory, the localization area needs to be divided into a number of grids. Nearly all the existing approaches simply treat the center of an identified grid as the target position, which would produce a large position error when the grid size is large.

## 3. Assumptions, Measurement Model and Motivations

### 3.1. Assumptions

The localization area is assumed to be two-dimensional (2*D*) plane and is divided into *N* equal grids which are the potential sites where the targets will be located. When the target appears in a grid, the center of the grid is represented as the target position. *K* targets are located in *K* different grids among the *N* grids. At every sampling time point, *M* APs measure the power/strength of the signals transmitted from all *K* targets and the *M* RSS measurements, one from each AP, are forwarded to the data fusion center to determine the positions of the *K* targets. The parameters *N* and *M* are the prior information, whereas *K* is unknown in advance. The relationship between the parameters satisfies M<<N, and K<<N.

### 3.2. Measurement Model

The Euclidean distance between the *m*-th AP and the target on the *n-*th grid is given by:

(1) $$d_{m,n}=\sqrt{{\left(x_{m}-x_{n}\right)}^{2}+{\left(y_{m}-y_{n}\right)}^{2}}\;\;1\leq m\leq M,1\leq n\leq N$$

where ($x_{m},y_{m}$) and ($x_{n},y_{n}$) are respectively the coordinates of the *m-*th AP and the center of the *n-*th grid. Usually, the RSS measurements at each AP are affected by obstructions, multipath propagation, and other indoor environmental factors. According to the indoor signal-fading model [43], when the signal is transmitted from the target at the *n*-th grid, the RSS measurement at the *m-*th AP is described as:

(2) $$p_{m,n}=p_{Tx}+G=p_{Tx}+G_{0}-n10\log(d)+X_{SSF}+X_{LSF}$$

where $P_{Tx}$ is transmitted signal power, G is the total power gain, d is the distance between the transmitter and receiver, $G_{0}$ is the path power gain in dBs at the reference distance and *n* is the path loss exponent, $X_{SSF}$ is the small-scale fading (SSF) contribution, i.e., the random variation of signal level due to multipath interference observed over one small-scale area, and $X_{LSF}$ is the large-scale fading (LSF) contribution, i.e., the random variation in local average of receiver power observed over a spatial extent of multiple small-scale areas.

Therefore, the total RSS measured at the *m-*th AP is then given by:

(3) $$u_{m}=\sum_{n=1}^{N}{Ρ}_{m,n}\vartheta_{n}+\varepsilon_{m}$$

where $\varepsilon_{m}$ is the measurement noise including modeling errors, $\vartheta_{n}$ is equal to one if a target is located in the *n*-th grid; otherwise, it is zero. Equation (3) can be written in a compact form as:

(4) u=Ρθ+ε

where:

(5) $$u=\left[\begin{array}{c} u_{1}\\ u_{2}\\ \vdots \\ u_{M} \end{array}\right],P=\left[\begin{array}{cccc} P_{1,1} & P_{1,2} & \cdots & P_{1,N}\\ P_{2,1} & P_{2,2} & \cdots & P_{2,N}\\ \vdots & \vdots & \vdots & \vdots \\ P_{M,1} & P_{M.2} & \cdots & P_{M.N} \end{array}\right],\theta =\left[\begin{array}{c} \vartheta_{1}\\ \vartheta_{2}\\ \vdots \\ \vartheta_{N} \end{array}\right],\varepsilon =\left[\begin{array}{c} \varepsilon_{1}\\ \varepsilon_{2}\\ \vdots \\ \varepsilon_{M} \end{array}\right]$$

Here θ is the unknown vector which has *K* ones and *N − K* zeroes. *N − K* would generally be much greater than *K*, so θ is a *K*-sparse vector. Therefore, the location problem can be considered as a *K*-sparse signal recovery issue which can be handled using the CS theory. Note that *u* is the actual measurement vector, whereas *P* is the theoretical measurement matrix.

Also note that in noise free condition, it can be seen that $\vartheta_{i}$ is equal to one or zero. If $\;\vartheta_{i}=1$, it means that the *i*-th grid has the target and corresponding center coordinate is the target’s position estimation. If $\;\vartheta_{i}=0$, it indicates that there is no target in the *i-*th grid.

### 3.3. Motivations

The presented work is motivated by the fact that a number of significant issues in localization have to be considered to make a localization algorithm more accurate and practical. Specifically, three motivations are associated with three issues as discussed below.

(1) The number of grids in a location area plays a significant role in target position estimation. Small number of grids will result in a larger position estimation error due to the large dimensions of a grid when treating the center of the grid as target position. Dividing the location area into smaller grids would improve position accuracy, but this would require more APs according to the CS theory, which may not be feasible in practice. Also, larger number of grids will lead to higher computational complexity. Therefore, this conflict should be handled in the development of the positioning algorithm.
(2) To employ the CS theory, it is necessary to have knowledge of the target population [39]. However, this assumption would typically not be true in reality. Thus, it is useful to develop an enhanced CS-based positioning algorithm without any prior knowledge of target population.
(3) As mentioned earlier, regardless of the grid size the center of the grid is usually chosen as the target position in the existing algorithms. However, the target can be at any place within the grid and such a selection may result in a considerable error especially when the dimensions of the grid are relatively large. Hence, it is important to develop methods to refine target positions especially when high accuracy position information is required. To cope with the above issues, a new two-phase CS-based positioning algorithm is proposed as described in the following section.

## 4. Proposed CS-Based Positioning Algorithm

**Figure 1:**
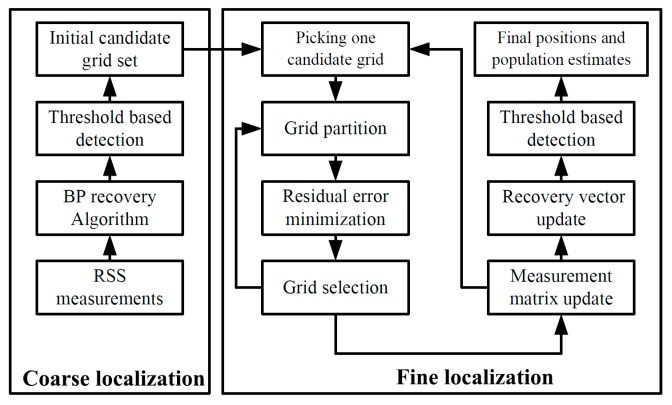
Block diagram of the proposed CS-based positioning scheme.

The block diagram of the proposed CS-based localization scheme for indoor environment is illustrated in Figure 1. The approach contains two main phases: (1) coarse localization and (2) fine localization.

In the coarse localization, the BP algorithm is employed to estimate the vector θ using the RSS measurements at the APs, which gives useful information to judge whether the grid has a target or not. The grids whose coefficients in θ are above a certain threshold are chosen as the candidate grids which have a target in each of them. That is, the number of the selected grids is the initial estimate of target population. This is different from the previous work [33,34,39], since the chosen grids are just the intermediate results rather than the final results.

In the fine localization, in order to reject the false grids and improve the position estimation accuracy, each of the initial candidate grids is partitioned iteratively for a pre-defined number of times and a smaller target grid is identified at every iteration time. As a consequence, the uncertainty of target position in the grid and the effect of grid size are significantly reduced. The recovery vector is then estimated again and threshold based detection is applied to determine the refined candidate grids and generate more accurate target population estimation. More details about the two phases are provided below.

### 4.1. Coarse Localization Phase

The main task of this phase is to find candidate grids where the targets may appear. Both the $\ell_{0}$-norm minimization and the $\ell_{1}$-norm minimization can be utilized to solve the target detection problem. However, the $\ell_{0}$-norm minimization is an NP-hard problem and hence the computational complexity is too high especially when *N* is large. The $\ell_{1}$-norm minimization is thus a better option to deal with the problem to generate an estimate of the sparse vector θ by:

(6) $$\hat{\theta }={\left[{\hat{\vartheta }}_{1},{\hat{\vartheta }}_{2},\cdots ,{\hat{\vartheta }}_{N}\right]}^{T}=\arg\underset{\theta }{\min\;}{\left\|\theta \right\|}_{1}\;\;s.t.\;\;||P^{T}(u-P\theta )|{|}_{\infty }<\sqrt{2\log N}\sigma$$

Note that two signal recovery algorithms can be employed to estimate θ, which are convex relaxation algorithm and greedy algorithm. One of the convex relaxation algorithms, called BP algorithm, is utilized to achieve the goal. Interested readers are referred to [44] for details of the convex optimization algorithm. For practical application, in order to obtain the exact reconstruction, it should obey the four-to-one rule which needs about four incoherent measurements per unknown nonzero term in reconstruction vector [29], i.e., *M > 4K*.

Based on the estimated *K*-sparse vector $\hat{\theta }$, the threshold based detection is exploited to choose the candidate target grids with the steps given below.

(1) A pre-defined threshold $\Delta_{1}$ is first determined such as based on conducting extensive simulations and data analysis. Certainly it would be useful to investigate on how to choose the threshold theoretically by allowing an acceptable false alarm probability in the future.
(2) If ${\hat{\vartheta }}_{i}\geq \Delta_{1},\;i=1,\;2,\;\cdots ,\;N$, the corresponding grids are assumed to hold the targets and the centers of the chosen grids are assumed to be the initial target’s position estimation. Their grid indexes are used to form the candidate grid index set $\Lambda_{1}$.
(3) If ${\hat{\vartheta }}_{i}<\Delta_{1}$, the corresponding grids are assumed not to have any target so that they are excluded from further processing.

Suppose that the size of the candidate grid index set $\Lambda_{1}$ is *L* which is much smaller than *N*. That is, the positions of the targets are supposedly constrained to these *L* grids. For convenience, according to the order in which they appear in the estimated sparse vector θ, the candidate grids are re-indexed to be from 1 to *L* based on the values of {${\hat{\vartheta }}_{i}$}. Also, the center position of the *i-*th grid is simply denoted by $\left(x_{i}^{\left(0\right)},\;y_{i}^{\left(0\right)}\right)$. Removing the elements whose values are below the threshold from the recovery signal vector $\hat{\theta }$ and using the re-arranged grid indexes produce a new recovery signal vector:

(7) $$\tilde{\theta }={\left[{\tilde{\vartheta }}_{1},\;{\tilde{\vartheta }}_{2},\;\cdots ,\;{\tilde{\vartheta }}_{L}\right]}^{T}$$

Accordingly, the dimensions of the new measurement matrix are reduced to *M* × *L*, which is defined as:

(8) $$\tilde{P}=[{\tilde{p}}_{1},\;{\tilde{p}}_{2},\;\cdots ,\;{\tilde{p}}_{L}]$$

Note that $\tilde{P}$ is a part of measurement matrix P whose *N – L* columns are removed to form $\tilde{P}$.

### 4.2. Fine Localization Phase

**Figure 2:**
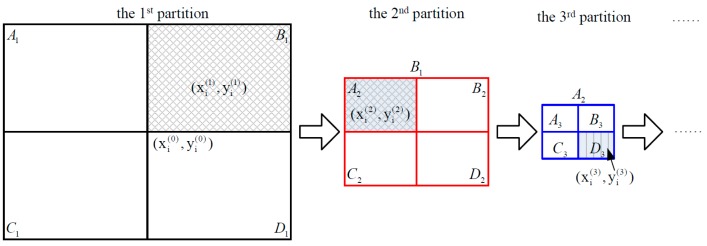
Schematic diagram of iterative partitioning of a grid.

Taking an arbitrary candidate grid with the center coordinates $\left(x_{i}^{\left(0\right)},\;y_{i}^{\left(0\right)}\right)$ as an example, from Figure 2, it can be seen that, at the 1st grid partition, the grid is divided into four equal-size grids *A*_1_, *B*_1_, *C*_1_, and *D*_1_. By defining the residual error as the squared difference between the measured RSS and the calculated one, the grid with the smallest residual error is selected as the target grid which is grid *B*_1_ in this case. Then, *B*_1_ is divided into four equal-size grids and the procedure continues for a pre-defined number of times. Then, the fine positioning phase is carried out in the following steps.

Before describing the steps, let us first initialize the residual measurement vector *r* and the intermediate measurement matrix Φ as r=u and Φ={}.

Step 1: Choose the candidate grid from the set $\Lambda_{1}$.

It is observed that the order of grid selection from $\Lambda_{1}$ for iterative partition affects localization performance considerably. Thus, a new method is proposed to solve the selection order problem. In the proposed algorithm, the desired candidate grid can be determined by:

(9) $$\hat{\lambda }=\arg\underset{\lambda \in \Lambda_{1}}{\max}\left|\langle r,{\tilde{p}}_{\lambda }\rangle \right|$$

where ${\tilde{p}}_{\lambda }$ is the column vector of the matrix $\tilde{P}$. λ is the index of the desired candidate grid. | | is the operation of absolute value and <, > is the operation of the usual inner product. 

Note that: the basic idea of the proposed grid selection algorithm is similar to the atom selection proposed in [45]. After the inner product calculation between the residual and each column of the measurement matrix, the column index with the maximum projection of the residual is chosen. The corresponding grid is considered as the desired candidate grid for grid partition.

Step 2: The selected gird is divided into four equal-size smaller grids. At the *j*-th iteration the target position can be represented as:

(10) $$(x_{\lambda }^{\left(j\right)},\;y_{\lambda }^{\left(j\right)})=(x_{\lambda }^{\left(j-1\right)},\;y_{\lambda }^{\left(j-1\right)})+\left({\left(-1\right)}^{\eta }\frac{d_{L}}{2^{j}},\;{\left(-1\right)}^{\zeta }\frac{d_{W}}{2^{j}}\right)$$

where $d_{L}$ and $d_{W}$ are the length and width of the initial grid, respectively and when treating the four equal-size grids as the four quadratures, one has:

(11) $$[\eta ,\;\zeta ]=\{\begin{array}{cc} {}[0,\;0], & \mathrm{First}\;\mathrm{quadrature}\\ {}[1,\;0], & \mathrm{Second}\;\mathrm{quadrature}\;\\ {}[1,\;1], & \mathrm{Third}\;\mathrm{quadrature}\\ {}[0,\;1], & \mathrm{Fourth}\;\mathrm{quadrature} \end{array}$$

After each grid partition, the potential target location area is reduced to a quarter of the previous location area. In theory the iterative partition can be realized as many times as possible and the target location area can be restricted to an extremely small area.

Next, according to (5), given the above four partitioned smaller grids, the theoretical measurement vector related to the *i*-th quadrature can be defined as:

(12) $$h_{\lambda }^{\left(i\right)}={\left[\begin{array}{cccc} p_{1,i} & p_{2,i} & \cdots & p_{M,i} \end{array}\right]}^{T},i=1,\;2,\;3,\;4$$

where $p_{j,i}$ is the theoretical signal power received by the *j-*th AP when the target transmitter is located at the center of the *i*th quadrature grid. The measurement matrix is then modified to be

(13) $${\tilde{P}}^{\left(i\right)}=\{\begin{array}{cc} {}[\begin{array}{cccc} h_{\lambda }^{\left(i\right)} & {\tilde{p}}_{2} & \cdots & {\tilde{p}}_{L} \end{array}], & \lambda =1\\ {}[\begin{array}{ccccccc} {\tilde{p}}_{1} & \cdots & {\tilde{p}}_{\lambda -1} & h_{\lambda }^{\left(i\right)} & {\tilde{p}}_{\lambda +1} & \cdots & {\tilde{p}}_{L} \end{array}], & 1<\lambda <L\\ {}[\begin{array}{cccc} {\tilde{p}}_{1} & \cdots & {\tilde{p}}_{L-1} & h_{\lambda }^{\left(i\right)} \end{array}], & \lambda =L \end{array}$$

Applying the least-square estimation produces the estimate of the recovery vector as:

(14) $${\hat{\theta }}^{\left(i\right)}={\left({\left({\tilde{P}}^{\left(i\right)}\right)}^{T}{\tilde{P}}^{\left(i\right)}\right)}^{-1}{\left({\tilde{P}}^{\left(i\right)}\right)}^{T}u$$

Then, based on the minimal residual error rule, the desired smaller candidate grid can be determined by:

(15) $$\hat{i}=\arg\underset{i\in \{1,2,3,4\}}{\min}{\left\|u-{\tilde{P}}^{\left(i\right)}{\hat{\theta }}^{\left(i\right)}\right\|}_{2}$$

The selected smaller grid is considered as the initial grid for next partition and the target position is updated by (10) based on which quadrature grid is selected.

Step 3: Implement the step 2 for the predefined number of iterative partition times *q*. The measurement vector of final selected smaller grid can be defined as:

(16) $$h_{\lambda }^{q}=h_{\lambda }^{\left(i\right)}$$

Step 4: Update the measurement matrix as:

(17) $$\tilde{P}=\{\begin{array}{cc} {}[\begin{array}{cccc} h_{\lambda }^{q} & {\tilde{p}}_{2} & \cdots & {\tilde{p}}_{L} \end{array}], & \lambda =1\\ {}[\begin{array}{ccccccc} {\tilde{p}}_{1} & \cdots & {\tilde{p}}_{\lambda -1} & h_{\lambda }^{q} & {\tilde{p}}_{\lambda +1} & \cdots & {\tilde{p}}_{L} \end{array}], & 1<\lambda <L\\ {}[\begin{array}{cccc} {\tilde{p}}_{1} & \cdots & {\tilde{p}}_{L-1} & h_{\lambda }^{q} \end{array}], & \lambda =L \end{array}$$

Then, update the intermediate measurement matrix as:

(18) $$\Phi =[\begin{array}{cc} \Phi , & {\tilde{p}}_{\lambda } \end{array}]$$

Applying least-squares estimation produces the estimate of the recovery vector as:

(19) $$\beta ={\left(\Phi^{T}\Phi \right)}^{-1}\Phi^{T}u$$

which is then used to calculate the update of the residual measurement vector:

(20) r=u−Φβ

So far, the processing of the selected grid is accomplished. The most possible position is obtained by (15) and the measurement matrix and residual measurement vector are updated by (16)–(20).

Step 5: Repeat steps 1–4 for *L* times so that all the *L* original candidate grids are iteratively partitioned for *q* times. The completely updated measurement matrix becomes

(21) $$\tilde{P}=[\begin{array}{cccc} h_{1}^{q} & h_{2}^{q} & \cdots & h_{L}^{q} \end{array}]$$

Step 6: Using the updated measurement matrix, the recovery vector can be re-estimated as:

(22) $$\hat{\theta }={\left({\tilde{P}}^{T}\tilde{P}\right)}^{-1}{\tilde{P}}^{T}u$$

The final target grids are determined by using the threshold based detection again. In this time, the threshold is set to be $\Delta_{2}$ and in the simulations the selection of the threshold will be discussed. The final target grids are those candidate grids whose recovery vector components are equal to or greater than the threshold, while the other candidate grids are discarded. As a consequence, the estimate of the target population is obtained and the positions of the targets are $\left\{(x_{k}^{\left(q\right)},\;y_{k}^{\left(q\right)})\},k\in \{1,\;2,\;\cdots ,\;L\right\}$ where the estimated target population is not greater than *L*.

### 4.3. Computational Complexity Analysis

For the proposed algorithm, since the BP recovery algorithm is utilized for coarse localization, the computational complexity in this phase is $\;O\left(M^{2}N^{3/2}\right)$. The fine localization phase uses the least squares estimation approach and thus the computational complexity is $\;O\left(ML^{2}\right)$, where M and L are the number of equations and number of variables, respectively. In the fine localization phase, the computational complexity in each grid partition (step 2 and step 3) is $O\left(4qML^{2}\right)$. The computational complexity in measurement matrix update (step 4) is $O\left(Mi^{2}\right)$. Thus, when the algorithm operates from step 1 to step 5, the computational complexity is $O\left(4qML^{3}\right)\;+\sum_{i=1}^{L}O\left(Mi^{2}\right)$. The computational complexity of step 6 is $O\left(ML^{2}\right)$. The total computational complexity of the proposed algorithm is $O\left(M^{2}N^{3/2}\right)+O\left(4qML^{3}\right)+\sum_{i=1}^{L}O\left(Mi^{2}\right)+O\left(ML^{2}\right)$. Thus, the computational complexity can be approximated to be $O\left(M^{2}N^{3/2}\right)+O\left(4qML^{3}\right)$. Table 1 shows the computational complexity comparison among different CS-based localization algorithms. Since the computational complexity of the OMP recovery technique is lower than the BP recovery technique, the computational complexity of the BP based localization algorithm is higher than that of OMP based localization algorithm. In the proposed algorithm, the BP recovery technique and the least squares estimation approach are utilized in the coarse localization phase and fine localization phase respectively. Thus, the proposed algorithm has the highest computational complexity among the above three localization methods.

## 5. Simulation Results

The location area is an 80 m × 80 m square region with *N* = 20 × 20 = 400 equal grids. The number of APs is assumed to be *M* = 121 for the simulation, which are deployed uniformly. The number of targets, *K*, is chosen from 2 to 7 to evaluate the effect of the target population [45] and their positions can be at any places of the given area rather than the center of the grid. The predefined threshold parameters are set to be $\Delta_{1}=0.15$ and $\Delta_{2}=0.3$. The number of iterative partitions is set at q=2 and the side length of the grid is *d* = 4 m.

The transmitted signal power is $p_{\mathrm{Tx}}=27\;\mathrm{dBm}$.$\;\;X_{SSF}$ is described by a mixture model for the probability density function $\alpha N\left(\mu_{\mathrm{dB}},\sigma_{\mathrm{dB}}\right)+\left(1-\alpha \right)\delta \left(X_{\mathrm{SSF}}\right)$, where N denotes a lognormal distribution with mean of the logarithmic Rice factor $\mu_{\mathrm{dB}}$, and standard deviation $\sigma_{\mathrm{dB}}$. $\delta \left(X_{\mathrm{SSF}}\right)\;\mathrm{is}$ the Dirac impulse function. α is the mixture weight. The above parameters can be defined by a polynomial fit function with χ, which are provided in Table 2. The path loss exponent *n* and the path power gain (in dBs at the reference distance) $G_{0}$ are modeled as Gaussian random variables with distribution parameters $N\left(\mu_{n},\sigma_{n}\right)$ and $N\left(\mu_{G0,\mathrm{dB}},\sigma_{G0,\mathrm{dB}}\right)$. $\rho_{\mathrm{nG}0,\mathrm{dB}}\;$ is the normalized correlation coefficient. $X_{LSF}$ has a log-normal distribution with the standard deviation $\sigma_{\mathrm{LSF},\mathrm{dB}}$. The distribution parameters of LSF environments are provided in Table 3. More details about the simulation parameters can be found in [43].

In the simulation, the performance of target population estimation, recovery estimation and the target position estimation are chosen for algorithm analysis. The mean and standard deviation are utilized to describe the performance of target population estimation. And the success rate and the false alarm rate are chosen to evaluate the signal recovery performance. The success rate is defined as the percentage that the distance between the actual target position and the estimated target position is less than the given distance D which can be adjusted. Meanwhile the false alarm rate is defined as the percentage that the distance between the estimated target position and the actual target position is larger than the given distance R which, in this paper, is set to be $2\sqrt{2}$ d. At last, the root mean square error (RMSE) and the error cumulative distribution function (CDF) are calculated as the positioning performance index. In this situation, occasional large position errors should be excluded for performance analysis, so the parameter D is the chosen as $2\sqrt{2}$ d which means that the estimated target position within the actual grid or adjacent grid is chosen. Also, 5% largest errors are also not used for calculation.

The GMP algorithm [42] is used for target population estimation performance comparison. Three OMP based algorithms (OMP clustering algorithm [34], the OMP weight clustering algorithm [33], and the OMP algorithm [45]) and GMP algorithm [42] are chosen for both the signal recovery estimation performance and positioning performance comparison. It should be noted that the three OMP based algorithms require that the target population *K* is known.

### 5.1. Effect of Parameter Selection

**Figure 3:**
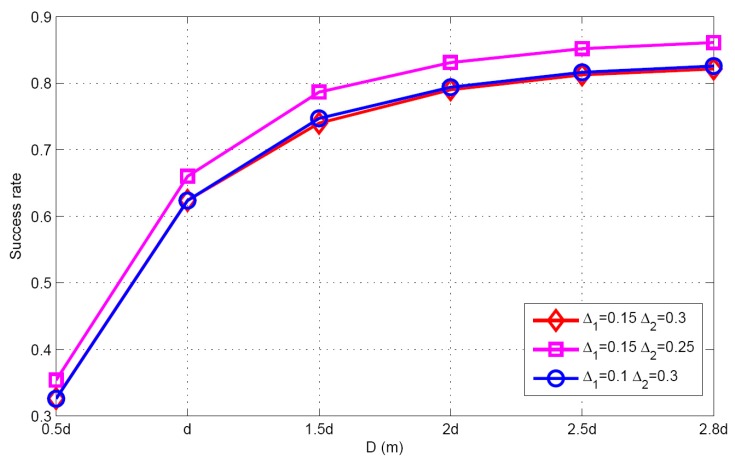
Success rate versus different thresholds.

**Figure 4:**
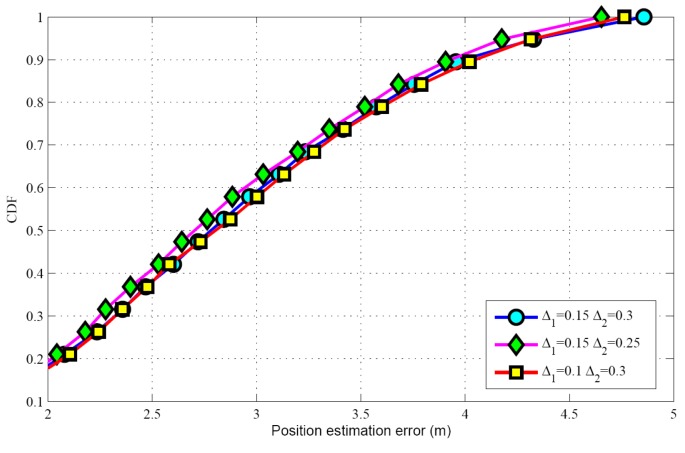
CDF versus different thresholds.

Taking SSF condition as an example, the performance under different thresholds are evaluated. The effects of the parameters such as target population and SNR can be found in the next section. Table 4 shows the target population estimation for different scenarios, when the actual target population *K* = 5. It can be seen that the threshold parameter $\Delta_{1}$ and $\Delta_{2}$ have a great effect on the target population estimation. When decreasing $\Delta_{1}$ or $\Delta_{2}$ in the proposed algorithm, the number of candidate grids will become larger. So the mean of target population estimation may be increased. Correspondingly, the success rate will be increased and the false alarm rate will be reduced. Figure 3 describes the success rate for different thresholds. Meanwhile, we can also see that increasing the threshold parameter $\Delta_{2}$ will lead to an increase in the estimated target population, so the mean and the success rate are also increased. Figure 4 illustrates the CDFs with different threshold parameters, showing that the threshold parameter has a minor impact on the position estimation.

### 5.2. Performance Comparison

#### 5.2.1. SSF Condition

**Figure 5:**
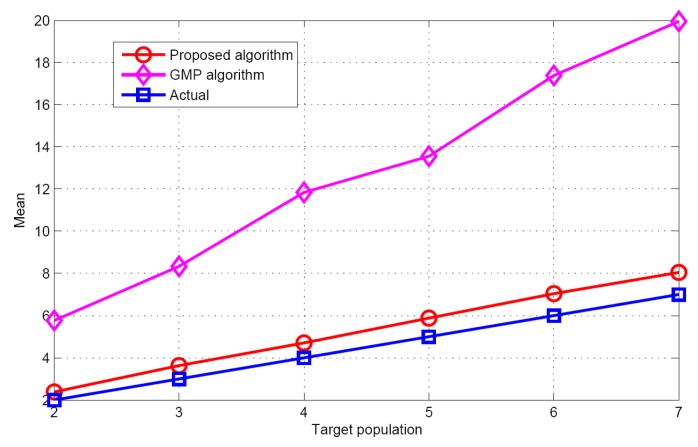
The mean of the estimated populations versus target population.

**Figure 6:**
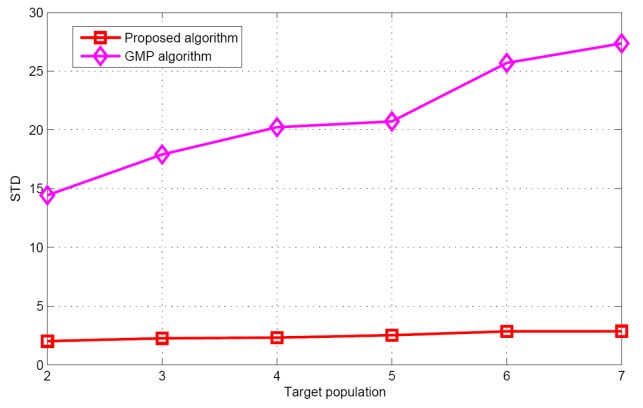
The STD of the estimated target populations versus target population.

Figure 5 and Figure 6 show the performance comparison of different target population estimation algorithms. It can be seen that as the target population increases, the performance of target population estimation will reduce. Meanwhile, the mean of the proposed algorithm is closer to the actual target population than that of GMP algorithm. The standard deviation of the proposed algorithm is also smaller than that of the GMP algorithm. For the GMP approach, the process of target population estimation is terminated, only when the residual error is stationary. However, in the real environments, the measurement vector contains many measurement errors such as measurement noise, modeling error etc. These measurement errors will have great effects on the target population estimation. Therefore, according to the simulation results and theoretical analysis, it can be concluded that regarding target population estimation, the proposed algorithm is better than the GMP algorithm.

**Figure 7:**
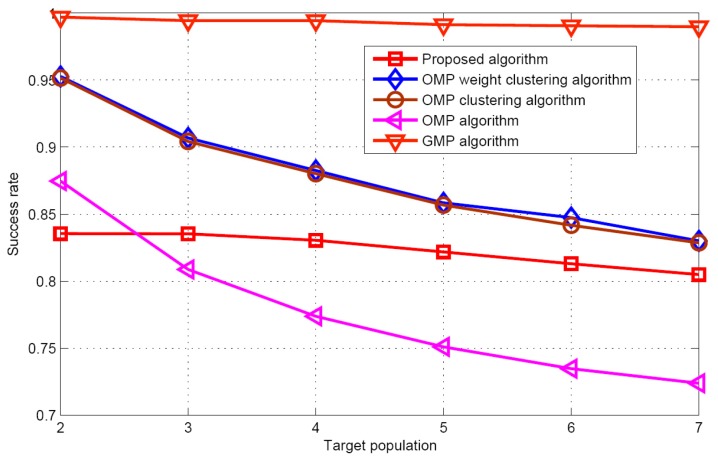
Success rate comparison (*D* = 2.8 d).

**Figure 8:**
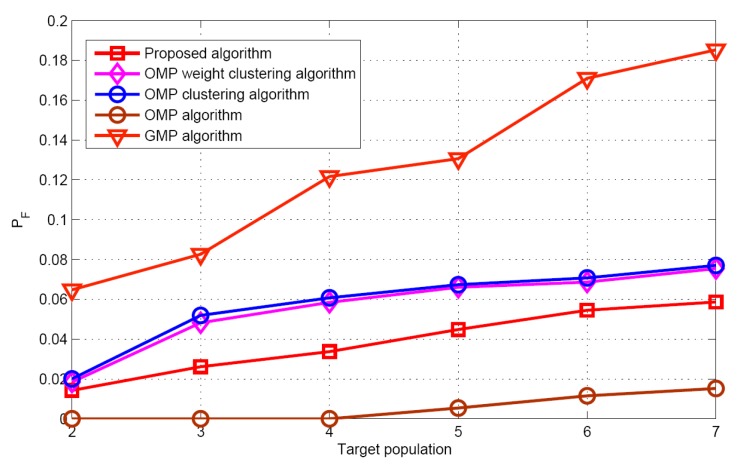
False alarm rate versus target population.

**Figure 9:**
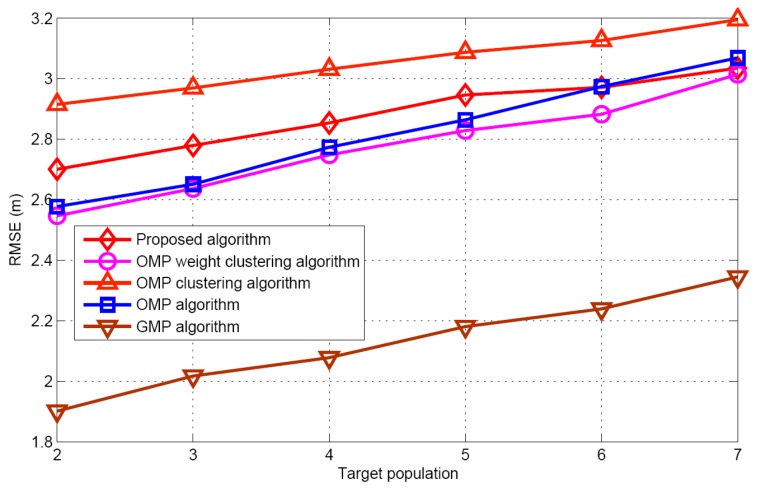
RMSE comparison versus target population.

Figure 7 and Figure 8 display the recovery performance comparison for *K* = 5 and SNR = 30 dB. As expected, the smaller the target population is, the better the recovery performance is. These results satisfy the CS theory itself. The larger target population estimation, leads to the best success rate and worst false alarm rate for the GMP algorithm. The OMP algorithm has the worst performance for success rate, because it does not consider the problem of the target position uncertainty in the grid. Although the proposed algorithm does not require any prior knowledge of the target population, it has similar performance as the OMP weight clustering approach and the OMP clustering approach, when the target population increases. Figure 9 describes the RMSE with respect to target population respectively for the five algorithms when SNR is 30 dB. Although the grid partition technique is used for the proposed algorithm and GMP algorithm. It can be seen that the performance of the GMP algorithm is much better than the proposed algorithm. The reason can be attributed that more number of target position estimation are obtained in the target population estimation.

**Figure 10:**
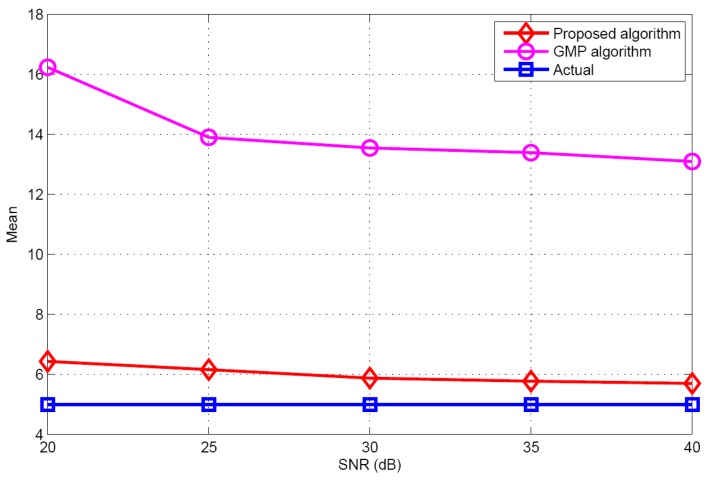
The mean of the estimated population versus SNR.

**Figure 11:**
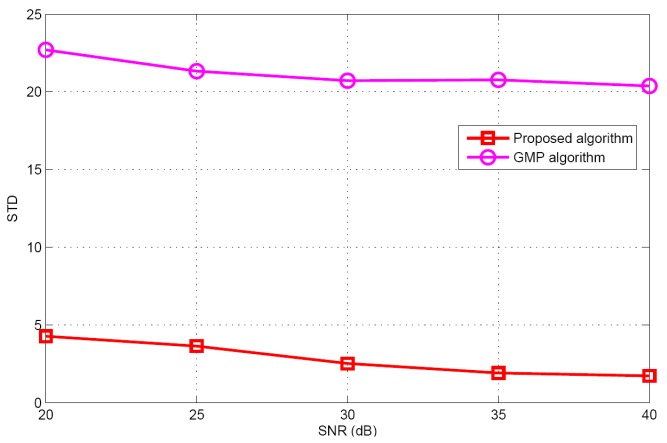
The STD of the estimated population versus SNR.

**Figure 12:**
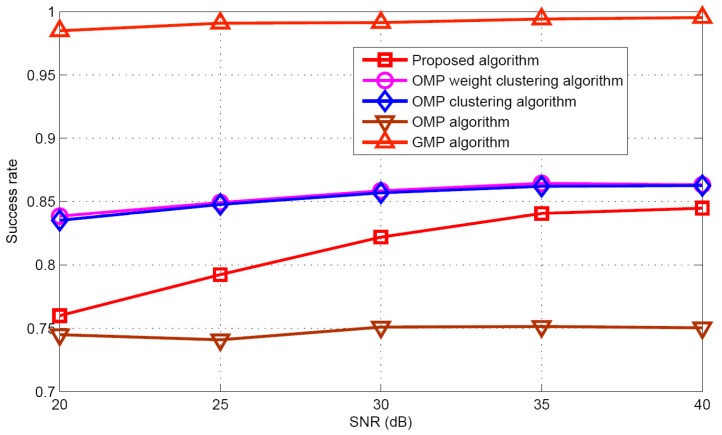
Success rate comparison (*D* = 2.8 d).

**Figure 13:**
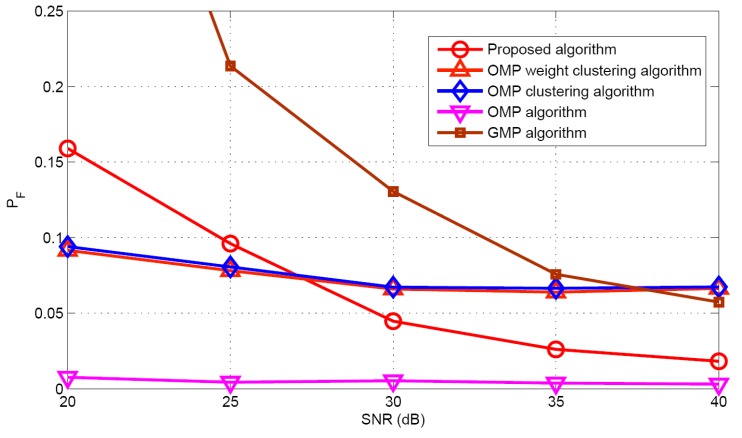
False alarm rate versus SNR.

**Figure 14:**
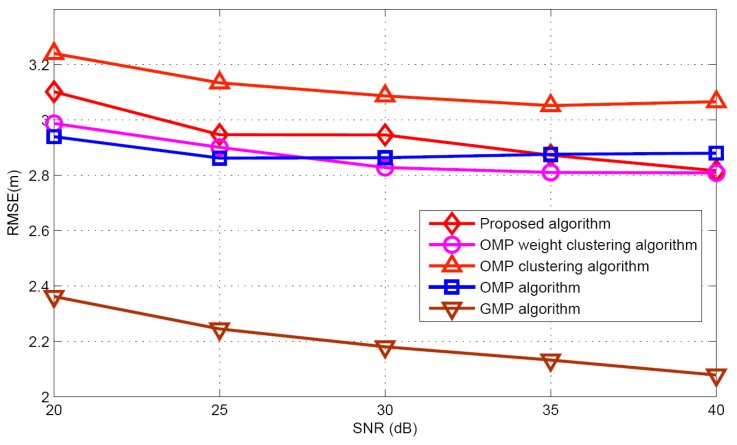
RMSE comparison versus SNR.

Taking target population *K* = 5 as an example, the performances of different algorithms are compared under different SNR conditions. Figure 10 and Figure 11 illustrate the mean and standard deviation of the target population estimation. It can be seen that as the SNR increases, the performance of target population estimation is basically improved. The proposed algorithm has better target population estimation performance than the GMP algorithm. Figure 12 and Figure 13 show the success rate and the false alarm rate of different algorithms. It can be seen that the performances of the proposed algorithm and the GMP algorithm are quite sensitive to SNR, whereas the OMP- based approaches are rather insensitive to SNR. Figure 14 describes the RMSE comparison with five algorithms. It can also see that the performance of GMP approach is best and the proposed algorithm has a similar localization performance as the other three algorithms.

To sum up, we can conclude that: (1) for target population estimation performance, the proposed algorithm performs much better than the GMP algorithm. Note that the other three OMP-based approaches should require the prior target population information (2) For signal recovery performance, the GMP algorithm has the best success rate and worst false alarm rate. The OMP algorithm has the worst success rate performance. The other three methods have a similar performance. (3) For positioning performance, the GMP algorithm is best and the other four approaches have a similar performance. Therefore, considering the above three performance indexes, the proposed algorithm is the best solution for practical applications.

#### 5.2.2. LSF Condition

**Figure 15:**
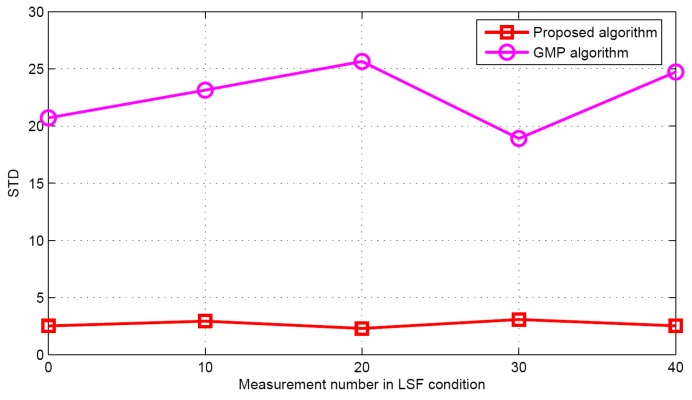
The mean of the estimated population in LSF conditions.

**Figure 16:**
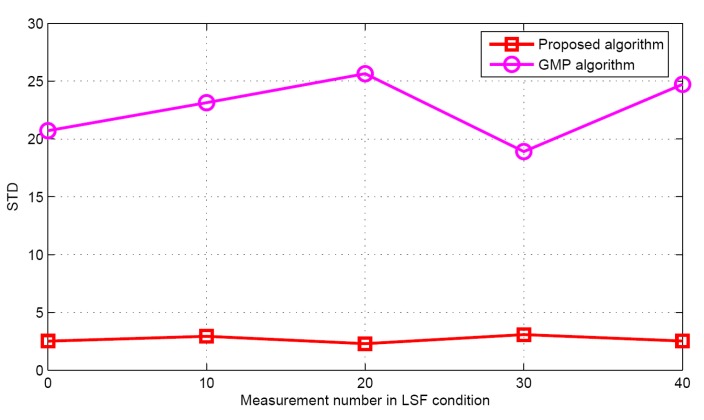
The STD of the estimated population in LSF conditions.

**Figure 17:**
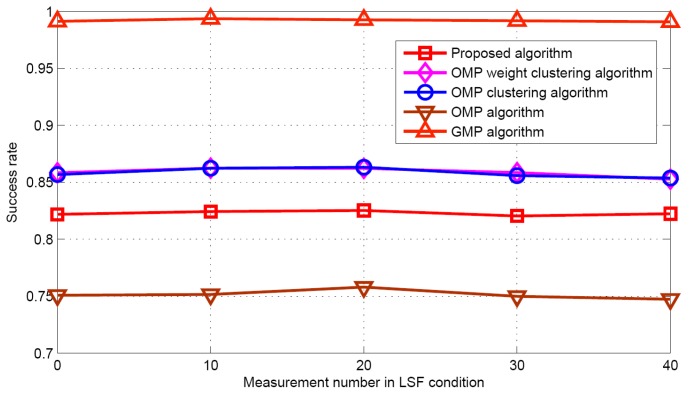
Success rate comparison.

**Figure 18:**
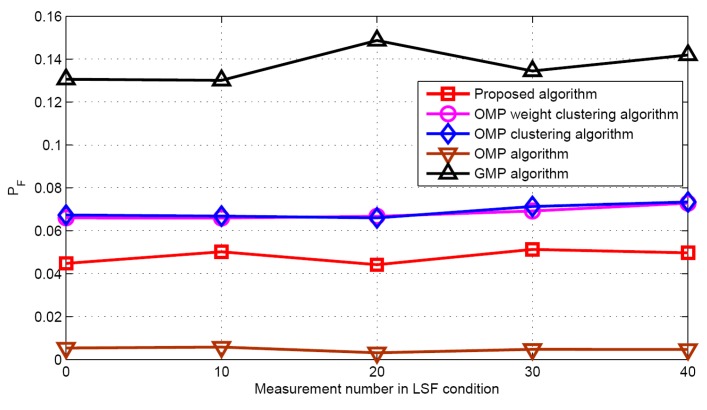
False alarm rate in LSF condition.

**Figure 19:**
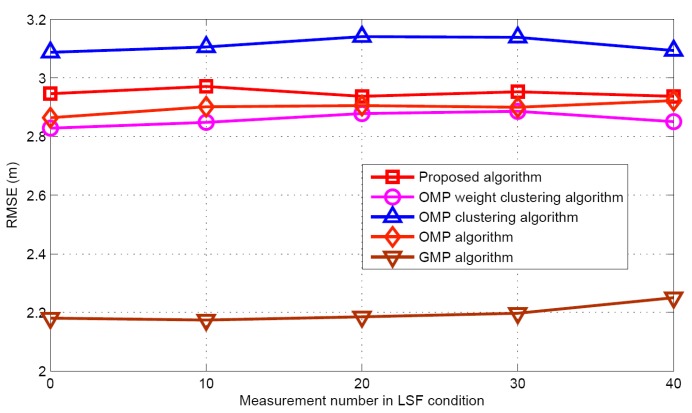
RMSE comparison in LSF conditions.

Now the performance comparisons of different algorithms are made with different measurement numbers under LSF conditions. The simulation parameters are *K* = 5 and SNR = 30 dB. Figure 15 and Figure 16 show the target population estimation performance (mean and standard deviation) of the proposed algorithm and the GMP algorithm. Figure 17 and Figure 18 illustrate the comparison of the success rate and that of the false alarm rate for the five algorithms, while Figure 19 displays the RMSE comparison when the measurement number in LSF condition is 25. It can be seen that same algorithm comparison conclusions can be obtained in the LSF condition. It should be noticed that since the CS technique has high recovery performance under some measurement noise conditions, all five approaches have a similar performance under different measurement numbers in LSF conditions.

## 6. Conclusions

In this article, a two-phase CS based target localization algorithm with RSS measurements has been proposed. This method does not require any prior knowledge of the target population, but it is able to estimate the target population. In the coarse localization phase, by considering localization as a signal recovery problem, initial candidate target grids are determined using the BP algorithm and the threshold-based detection technique. In the fine localization phase, by iterative grid partition and measurement matrix update, target position can be constrained to a sufficiently small area so that the target position uncertainty in a grid is greatly reduced. Compared to other existing approaches, the proposed algorithm can be considered as a best solution for practical applications with respect to target population estimation, signal recovery estimation and position estimation.

## Figures and Tables

**Table 1:** Computational complexity comparison.

Algorithm	Computational Complexity
BP-based localization algorithm	$o\left(M^{2}N^{3/2}\right)$
OMP-based localization algorithm	o(KMN)
Proposed algorithm	$O\left(M^{2}N^{3/2}\right)+O\left(4qML^{3}\right)$

**Table 2:** Parameters for SSF environment.

$\mu_{dB}$				$\sigma_{dB}$	α	
$p\left(\chi \right)=c_{3}\chi^{3}+c_{2}{\;\chi }^{2}+c_{1}\chi +c_{0}$				$p\left(\chi \right)=b_{0}$	$p\left(\chi \right)=a_{1}\chi +a_{0}$	
$c_{3}$	$c_{2}$	$c_{1}$	${\;c}_{0}$	$b_{0}$	$a_{1}$	$a_{0}$
1.23	−9.52	20.64	−8.17	3.84	−0.05	1.05

In Table 2, χ is the distance between transmitter and mid-point of modeled SSA (m).

**Table 3:** Parameters in *G*_db_(d) for LSF environment.

$\mu_{n}$	$\sigma_{n}$	$\mu_{G0,dB}\;\;$	$\sigma_{G0,dB}$	$\rho_{nG0,dB}$	$\sigma_{LSF,dB}$
2.5	0.3	−50.9	2.7	0.1	1.5

**Table 4:** The results for robust tests.

	$\Delta_{1}\;$= 0.15 $\Delta_{2}\;$= 0.3	$\Delta_{1\;}$= 0.15 $\Delta_{2}\;$= 0.25	$\Delta_{1}\;$= 0.1 $\Delta_{2}\;$= 0.3
Mean	5.8790	6.5905	5.9285
Std	2.5166	2.6884	2.5696
False alarm rate	0.0448	0.0673	0.0479
